# A Rare Primary Pituitary Abscess Caused by Cutibacterium Acnes

**DOI:** 10.1055/a-2641-6415

**Published:** 2025-07-12

**Authors:** Chi-Man Yip

**Affiliations:** 1 Division of Neurosurgery, Kaohsiung Veterans General Hospital, Kaohsiung, Taiwan

**Keywords:** cutibacterium acnes, hypopituitarism, primary pituitary abscess, rim-like enhanced sellar lesion

## Abstract

**Introduction:**

Pituitary abscess is a rare but potentially life-threatening condition with an incidence of 0.2 to 1.1% of operative pituitary lesions. Preoperative diagnosis is difficult because it shares many similarities with other pituitary lesions in terms of signs and symptoms and radiographic findings. The author would like to share a case of primary pituitary abscess due to Cutibacterium acnes infection, which is probably the first case reported in an adult patient.

**Case Presentation:**

A 60-year-old woman with having medical history of diabetes mellitus who suffered from severe headache, fever, chillness, and vomiting in January 2024. She had been admitted to the Infectious Diseases Department; however, no definite infection source was found, but hypopituitarism was detected. Her brain magnetic resonance imaging (MRI) showed a rim-like enhanced sellar lesion with suprasellar extension. She underwent an endoscopic endonasal transsphenoidal approach with the removal of the lesion and skull base reconstruction. During the surgery, pus-like material and some solid tissue, which was yellowish white in color, were found. The culture of the pus revealed the growth of Cutibacterium acnes, and the histological report of the solid tissue proved nonneoplastic pituitary gland tissue, admixed with fibrous tissue and marked chronic inflammation. She recovered well after surgery and completed antibiotic treatment.

**Conclusion:**

Preoperative diagnosis of pituitary abscess is difficult. The majority of pituitary abscesses are diagnosed during the operation or postoperatively. Prompt diagnosis and treatment of pituitary abscess yield a favorable prognosis. The mainstay of treatment is transsphenoidal surgical resection in combination with antibiotic therapy.

## Introduction

Pituitary abscess is a rare but potentially life-threatening condition with an incidence of 0.2 to 1.1% of operative pituitary lesions. 1 2 In 1848, Heslop reported the first case of pituitary abscess. 3 Up to 2023, there were fewer than 500 cases of pituitary abscess reported. 4 Preoperative diagnosis is difficult because pituitary abscess shares many similarities with other pituitary lesions in terms of signs and symptoms and radiographic findings. The author would like to share a case of primary pituitary abscess with positive culture of Cutibacterium acnes. To our best knowledge, this is probably the third reported case of primary pituitary abscess due solely to Cutibacterium acnes infection. However, in adult patients, it is probably the first reported Cutibacterium acnes primary pituitary abscess, because the previous two reported cases were pediatric patients.

## Case Presentation

**Fig. 1:**
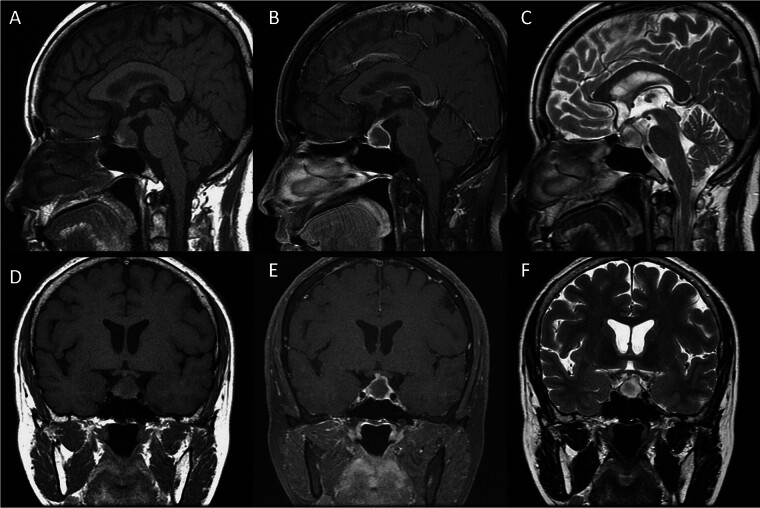
Preoperative brain MRI. Sagittal T1WI ( **A** ), sagittal T1WI post Gadolinium injection ( **B** ), sagittal T2WI ( **C** ), coronal T1WI ( **D** ), coronal T1WI post Gadolinium injection ( **E** ), coronal T2WI ( **F** ) demonstrated a lesion hypointense in T1-weighted imaging, hyperintense in T2-weighted imaging and had rim-like enhancement after gadolinium injection over the sella and suprasellar region.

A 60-year-old woman with having medical history of diabetes mellitus since 2019, who suffered from severe headache, fever, chillness, and vomiting in January 2024, approximately 3 months prior to her presentation to our Neurosurgery service. She did not have sinus surgery, cranial surgery, or dental procedures in the past. She had been admitted to the Infectious Diseases Department; however, no definite infection source was identified; her laboratory data, including C-reactive protein, were within normal limits, but low levels of cortisol and free T4 were detected (cortisol = 1.01 μg/dL, free T4 = 0.52 ng/dL). Brain MRI was arranged for further evaluation of her recurrent chill sensation, fatigue, and less energy. Her brain MRI showed a round lesion approximately 1.9 cm × 1.6 cm × 1.6 cm in size located at the sella and suprasellar region, which was hypointense in T1-weighted imaging, hyperintense in T2-weighted imaging and had rim-like enhancement after gadolinium injection, and this lesion showed attachment to the optic chiasma ( Fig. 1 ). Neurosurgeon was then consulted, and she was transferred to neurosurgery service. Her neurological examination and physical examination were essentially normal, except her visual acuity showed impairment.

**Fig. 2:**
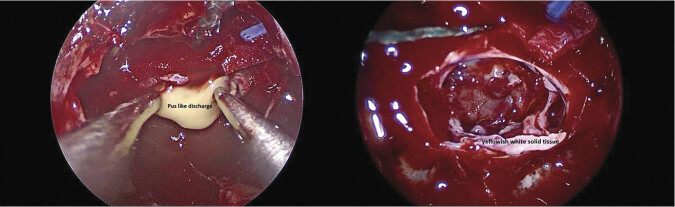
Intraoperative pictures. Pus-like material and some solid tissue, which was yellowish white in color, were found.

**Fig. 3:**
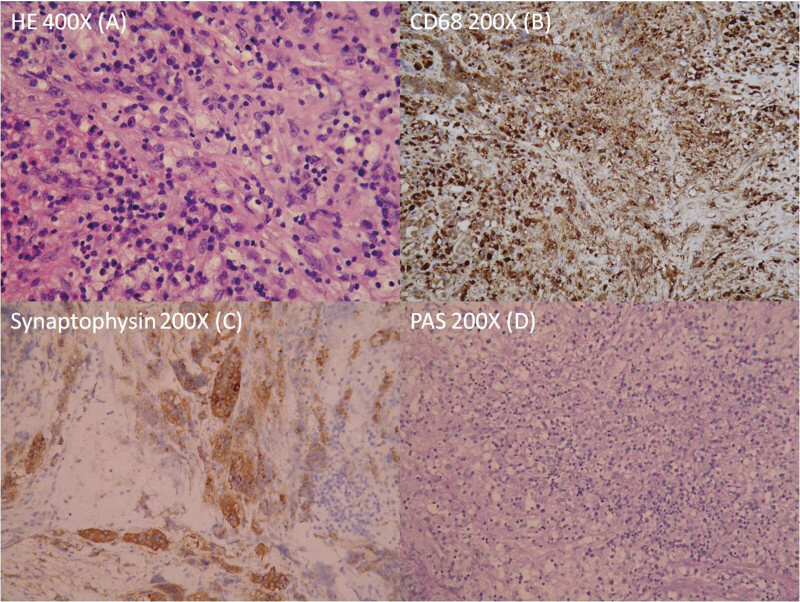
Histology of the solid tissue. ( **A** ) Showed marked chronic inflammatory cells, including foamy histiocytes, small lymphocytes, and plasma cells. ( **B** ) Showed the presence of macrophages and monocytes. ( **C** ) Demonstrated the presence of some nonneoplastic pituitary gland tissue. ( **D** ) No fungus was seen.

After a thorough discussion with the patient and thorough preoperative evaluation, under the impression of pituitary macroadenoma with cystic/necrotic change, surgical intervention was arranged. Under general anesthesia, she underwent an endoscopic endonasal transsphenoidal approach with the removal of the lesion and skull base reconstruction with a free mucosal graft harvested from the resected right middle turbinate. During the surgery, pus-like material and some solid tissue, which was yellowish white in color, were found ( Fig. 2 ); they were removed for culture and histology examination. The culture of the pus revealed the growth of Cutibacterium acnes. Histology examination of the solid tissue showed marked chronic inflammatory cells, including foamy histiocytes, small lymphocytes, and plasma cells in hematoxylin and eosin (HE) stain. Immunohistochemistry revealed the presence of macrophages and monocytes in CD68 staining, the presence of some nonneoplastic pituitary gland tissue in Synaptophysin staining, and no fungus was detected in PAS staining. Based on the results of HE stain and immunohistochemical stains, the excised solid tissue proved to be nonneoplastic pituitary gland tissue, admixed with fibrous tissue and marked chronic inflammation ( Fig. 3 ).

**Fig. 4:**
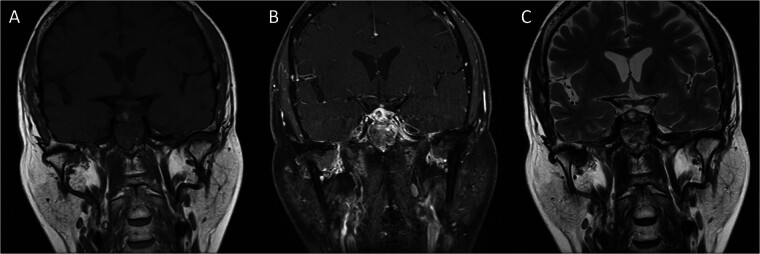
Postoperative brain MRI. Coronal T1WI ( **A** ), coronal T1WI post Gadolinium injection ( **B** ), coronal T2WI ( **C** ) showed the regression of the rim-like enhanced lesion over the sella and suprasellar region.

After the diagnosis of primary pituitary abscess was established, an infectious physician was consulted and intravenous antibiotics with Ceftriaxone were prescribed and lasted for 4 weeks, then she was discharged home with oral antibiotics, Amoxicillin, which lasted for 9 weeks as recommended by the infectious physician. This patient recovered well; her vision improved; her headache was gone, but her hypopituitarism was still present. She is regularly followed up at the endocrinology outpatient department and is under regular hormone replacement. Her sellar MRI was followed 2 months after the surgery, which showed regression of the rim-like enhancement over the sella and suprasellar region ( Fig. 4 ).

## Discussion

The differential diagnosis of a rim-like enhanced lesion in the sellar region includes pituitary adenoma with cystic/necrotic change, Rathke's cleft cysts, craniopharyngioma, pituitary abscess, or other lesion entities. 5 Typical radiological features of pituitary abscess on MRI are space space-occupying lesion in the sellar region, having hypointense on T1-weighted imaging and hyperintense on T2-weighted imaging, with rim enhancement after gadolinium injection. 4 5 6 Pituitary abscesses are classified as primary or secondary. 1 2 4 Primary pituitary abscesses are caused by hematogenous pathogen spread or by direct extension of an adjacent infection to a normal pituitary gland. 1 2 4 Secondary pituitary abscesses are secondary infections of preexisting sellar lesions with or without a recent history of surgical treatment. 1 2 4 From the literature, primary pituitary abscesses are more common than secondary pituitary abscesses. 1 2 4 6 Clinical presentation of pituitary abscess may be quite variable, ranging from paucisymptomatic cases to severe compartment syndrome with mass effect on pituitary gland and/or optic apparatus, hence causing different degrees of endocrine disorders and visual disturbances. 1 The most common clinical presentations are headache, visual impairment, and endocrine abnormalities. 2 5 6 7 The presence of diabetes insipidus can make clinicians more alert to diagnose pituitary abscess. 1 5 Functional recovery is good for visual disturbances and headache, but pituitary function recovery remains poor. 1 Preoperative diagnosis of pituitary abscess is difficult. The majority of pituitary abscesses are diagnosed during the operation or postoperatively. Prompt diagnosis and treatment of pituitary abscess yield a favorable prognosis. The mainstay of treatment is surgical intervention, either through a transsphenoidal approach or craniotomy approach, in combination with adequate and proper antibiotic therapy and hormone replacement if indicated. 2 Pituitary abscess can be fatal; the reported mortality rate ranges from 4.5 to 8% based on different series, and the causes of death are due to pituitary dysfunction and/or spread of infection with resulting sepsis. 2 4

Not all cases yield positive pathogen culture; the positive rates for cultures ranged from 0 to 64% based on different series. 5 The most common causative bacteria are Staphylococcus species and Streptococcus species, 1 2 4 while Aspergillus is the most common isolated fungal organism. 2 4 Shuster et al and Sherrod et al had reported pituitary abscess caused by Cutibacterium acnes infection in 2010 and 2021, respectively, and their patients were pediatric patients. 8 9 Back to this particular patient, she is probably the first adult patient having a pituitary abscess proven to be infected by Cutibacterium acnes. With the collaboration of a neurosurgeon, infectious physician, and endocrinologist, this particular patient had a favorable outcome.

Cutibacterium acnes is an anaerobic, gram-positive bacterium of the normal flora of the skin, hair follicles, sebaceous glands, oral cavity, genitourinary and gastrointestinal tracts, conjunctiva, and the external ear canal. 10 11 This commensal bacterium could become an opportunistic pathogen, but the underlying processes are unclear. 10 The author supposes that diabetes mellitus may be the risk factor for this particular patient to get this unusual infection. Cutibacterium acnes is sensitive to penicillin, vancomycin, and cephalosporins, with vancomycin showing the greatest bactericidal effect. 11 Biomarkers that characterize Cutibacterium acnes infection have not yet been identified. Classical biomarkers of bacterial infection, such as C-reactive protein, erythrocyte sedimentation rate, or procalcitonin, have shown limited utility in diagnosing Cutibacterium acnes infections. 10 New diagnostic methods are needed.

## Conclusion

Preoperative diagnosis of pituitary abscess is difficult. The majority of pituitary abscesses are diagnosed during the operation or postoperatively. The mainstay of treatment is transsphenoidal surgical intervention to obtain tissue histological diagnosis and pathogen culture data, plus proper antibiotic therapy based on the pathogen detected, if possible. Prompt diagnosis and treatment of pituitary abscess yield a favorable prognosis; however, pituitary abscess can be fatal; clinicians should be alerted to this uncommon but benign disease. Collaboration of a neurosurgeon, infectious physician, and endocrinologist is essential to treat pituitary abscess patients.
